# A Functional Agonist of Insect Olfactory Receptors: Behavior, Physiology and Structure

**DOI:** 10.3389/fncel.2019.00134

**Published:** 2019-04-29

**Authors:** Srishti Batra, Jacob Corcoran, Dan-Dan Zhang, Pramit Pal, Umesh K.P., Renuka Kulkarni, Christer Löfstedt, Ramanathan Sowdhamini, Shannon B. Olsson

**Affiliations:** ^1^National Centre for Biological Sciences, Tata Institute of Fundamental Research, Bengaluru, India; ^2^Department of Biology, Lund University, Lund, Sweden

**Keywords:** olfaction, olfactory receptors, electrophysiology, behavior, molecular docking, heterologous expression

## Abstract

Chemical signaling is ubiquitous and employs a variety of receptor types to detect the cacophony of molecules relevant for each living organism. Insects, our most diverse taxon, have evolved unique olfactory receptors with as little as 10% sequence identity between receptor types. We have identified a promiscuous volatile, 2-methyltetrahydro-3-furanone (coffee furanone), that elicits chemosensory and behavioral activity across multiple insect orders and receptors. *In vivo* and *in vitro* physiology showed that coffee furanone was detected by roughly 80% of the recorded neurons expressing the insect-specific olfactory receptor complex in the antenna of *Drosophila melanogaster*, at concentrations similar to other known, and less promiscuous, ligands. Neurons expressing specialized receptors, other chemoreceptor types, or mutants lacking the complex entirely did not respond to this compound. This indicates that coffee furanone is a promiscuous ligand for the insect olfactory receptor complex itself and did not induce non-specific cellular responses. In addition, we present homology modeling and docking studies with selected olfactory receptors that suggest conserved interaction regions for both coffee furanone and known ligands. Apart from its physiological activity, this known food additive elicits a behavioral response for several insects, including mosquitoes, flies, and cockroaches. A broad-scale behaviorally active molecule non-toxic to humans thus has significant implications for health and agriculture. Coffee furanone serves as a unique tool to unlock molecular, physiological, and behavioral relationships across this diverse receptor family and animal taxa.

## Introduction

Chemical signaling is the most ancient form of communication on Earth, and all forms of life use chemicals to communicate with each other and their environment (Eisner and Meinwald, 1995). Transmembrane protein chemoreceptors are employed by organisms to detect these chemical signals, and represent a wide variety of protein types such as receptor kinases or cyclases, transient receptor potential channels, ionotropic receptors, and others (Wicher and Große-Wilde, 2017). Olfactory receptors are a type of chemoreceptor expressed on the dendrites of olfactory sensory neurons in most animals, and were first characterized in vertebrates (Buck and Axel, 1991) as a family of seven-transmembrane, G protein-coupled receptors (GPCRs).

Despite bearing the same name as their vertebrate counterpart, insect olfactory receptors (Ors; Clyne et al., 1999; Gao and Chess, 1999; Vosshall et al., 1999) are not canonical GPCRs, but exhibit an inverted topology (Benton et al., 2006; Butterwick et al., 2018; Luo and Carlson, 2018), and share very low sequence identity even with close species (Benton, 2015). Insect Ors also form a heteromeric ion channel comprising an odor-specific protein and a co-receptor known as Orco, which is common to all flying insects (Larsson et al., 2004). Volatile ligands interact with this complex leading to influx of calcium and neuronal activation of olfactory sensory neurons (OSNs). Each receptor type binds to a specific subset of chemical ligands. Within insect species, the same ligand can interact with several Ors, and these same Ors can interact with diverse odorants, establishing a combinatorial code for odor identity across the receptor repertoire (Malnic et al., 1999; De Bruyne et al., 2001; Hallem et al., 2004; Hallem and Carlson, 2006; Benton, 2015; Wicher and Große-Wilde, 2017).

Coupled with the unusual topology of the insect olfactory receptor complex, this diverse family creates an apparent paradox: how can these unique proteins simultaneously bind to both similar and diverse ligands with such selectivity? To date, we have very little understanding of how this highly divergent gene family serves as a “selectively non-selective” (Berna et al., 2009) system for detecting odorants. One way to tackle this question is to identify a ligand that binds to a wide variety of these receptors, and compare its binding properties across receptors. While each insect species has evolved a unique repertoire of olfactory receptors for their specific ecological needs, no natural ligand is known that can activate diverse receptor types across different species (Münch and Galizia, 2016). Unfortunately, the only ligand known to activate the OrX-Orco complex to date is a non-volatile synthetic molecule VUAA1 (Jones et al., 2011), which acts on the conserved Orco subunit, providing little information for the divergent olfactory receptor family itself. In this study, we report a promiscuous natural volatile, 2-methyltetrahydrofuran-3-one, known as coffee furanone, which activates the antenna of several insect orders, including most olfactory sensory neurons in *Drosophila melanogaster*. Electrophysiological and heterologous expression studies show that this volatile activates sensory neurons through the variable “OrX” unit of the OrX-Orco complex. The promiscuity of this ligand across the insect olfactory receptor family allowed us to then model three-dimensional structures and perform docking studies with the ligand on selected Ors. Using these methods, we propose a conserved motif and extracellular loop region we predicted to serve as a base for ligand binding for insect olfactory receptors. Finally, bioassays indicate that coffee furanone elicits attraction and repellence across multiple insect taxa.

## Results

**FIGURE 1:**
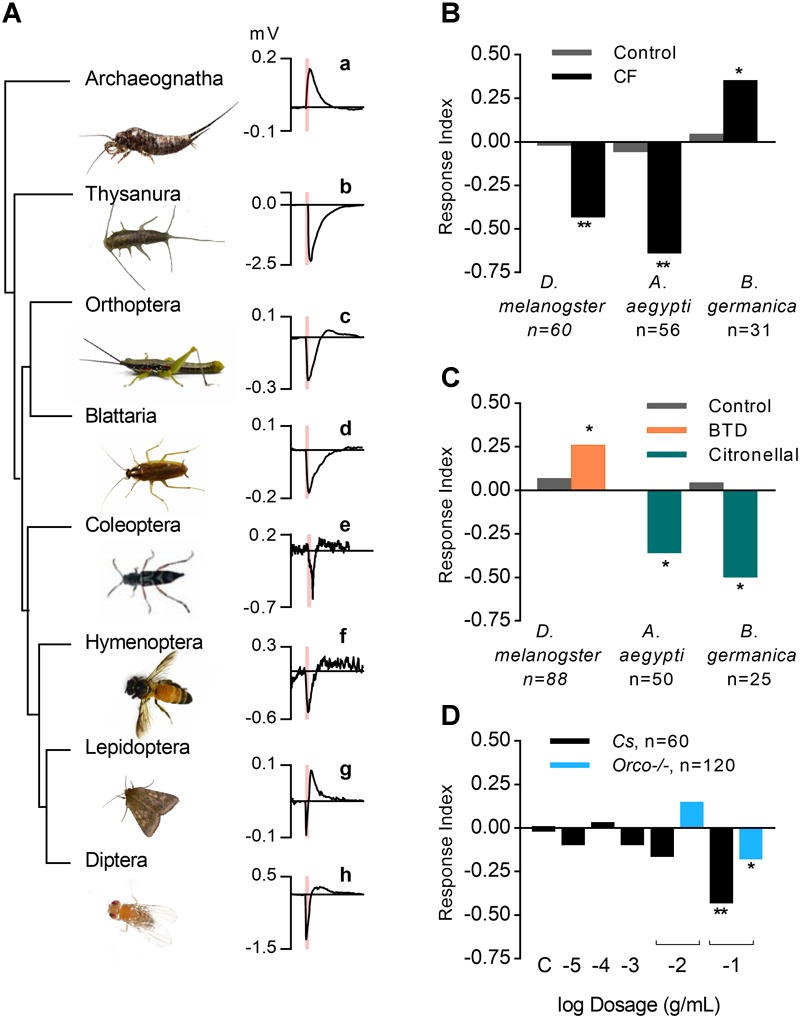
Electrophysiological and behavioral responses evoked by coffee furanone for different insect orders. **(A)** Left: phylogeny of insects for which coffee furanone (CF) elicited antennal activity. Right graphs indicate electroantennogram responses of respective species to 0.5 s odor stimulations of coffee furanone (red bar) at concentration of 10^-1^ g/mL and 0.5 L/min. **a**, *Lepismachilis γ-signata* (image from Missbach et al., 2014), **b**, silverfish **c**, grasshopper **d**, *Blattella germanica* **e**, *Xylotrechus quadripes* **f**, *Apis dorsata* **g**, *Helicoverpa armigera* **h**, *Drosophila melanogaster*. **(B)** *Drosophila melanogaster, CantonS (Cs)* strain, *Blattella germanica* tested at 10^-1^ g/mL dilution, and *Aedes aegypti* tested with 10^0^ g/mL of CF in Y-tube olfactometry assays (CF = coffee furanone; ^∗∗^*p* value < 0.005, ^∗^*p* value < 0.05, Chi-square test). **(C)** *Drosophila melanogaster, CantonS (Cs)* strain tested to known attractant 2, 3–butanedione (BTD) at a concentration of 10^-2^ g/mL; *Aedes aegypti* and *Blattella germanica* tested to known repellent citronellal at 10^-2^ g/mL. **(D)** *Cs* and Orco-/- flies (C = control), tested for various concentrations of coffee furanone.

To investigate the chemosensory properties of coffee furanone, we first carried out electroantennogram analyses across several orders of insects (Figure 1A). All flying orders of insects tested responded to this volatile, and are also known to express the insect OrX-Orco complex (Missbach et al., 2014; Brand et al., 2018). Silverfish, which belong to the ancient, nonflying order Zygentoma, are known to have Ors and Orco in their genome (Brand et al., 2018), and likewise responded in a similar fashion to flying insect orders that contain the full Or-Orco complex [Figure 1A(b)]. Nevertheless, the antennal response in bristletails, *Lepismachilisγ-signata*, a species from the most basal insect order Archaeognatha and known to lack the complex entirely (Missbach et al., 2014), exhibited a reversed polarity [Figure 1A(a)] similar to Orco-/- flies, where Or-expressing OSNs are silenced (Supplementary Figure 1). This provided an early indication for the importance of the Or-Orco complex in the olfactory response. Please note that such reversed polarity responses have also been previously reported in other studies as well (e.g., Krång et al., 2012), and the deflection from the baseline implies that other receptor types on the antenna in addition to the Or-Orco complex could also be involved in detection of these molecules.

When tested in behavioral assays, coffee furanone was repellent in *Drosophila melanogaster* and *Aedes aegypti*, while it was attractive to cockroaches, *Blattella germanica* (Figure 1B). To test that the observed behavioral activity was not due to the concentrations used, we also tested known attractants and repellents for these species as positive controls (Figure 1C) (Cockcroft et al., 1998; Knaden et al., 2012). This behavioral activity was altered in Orco-/- flies (Figure 1D) where mutants showed reduced repellence at both 10^-2^ g/mL (Response index = 0.15 for Orco-/- and RI = -0.167 for *Cs*) and 10^-1^ g/mL (RI = -0.18 for Orco-/- and RI = -0.433 for *Cs*), providing further evidence of the importance of the Or-Orco complex for chemosensory detection of this compound.

To confirm the molecular target(s), we performed single sensillum electrophysiology analyses in *Drosophila melanogaster*, one of the only insect species with an elucidated chemosensory repertoire (Olsson et al., 2015; Münch and Galizia, 2016). *D. melanogaster* and other flying insects express three major types of chemosensory proteins on the dendrites of antennal sensory neurons: olfactory receptors (Ors), gustatory receptors (Grs), and ionotropic receptors (Irs) (Stocker, 1994; Vosshall and Stocker, 2007; Benton et al., 2009). While antennal Grs may stimulate ion channels directly (Sato et al., 2011) or indirectly (Badsha et al., 2012), both Irs and Ors form ion channels themselves (Wicher and Große-Wilde, 2017).

**FIGURE 2:**
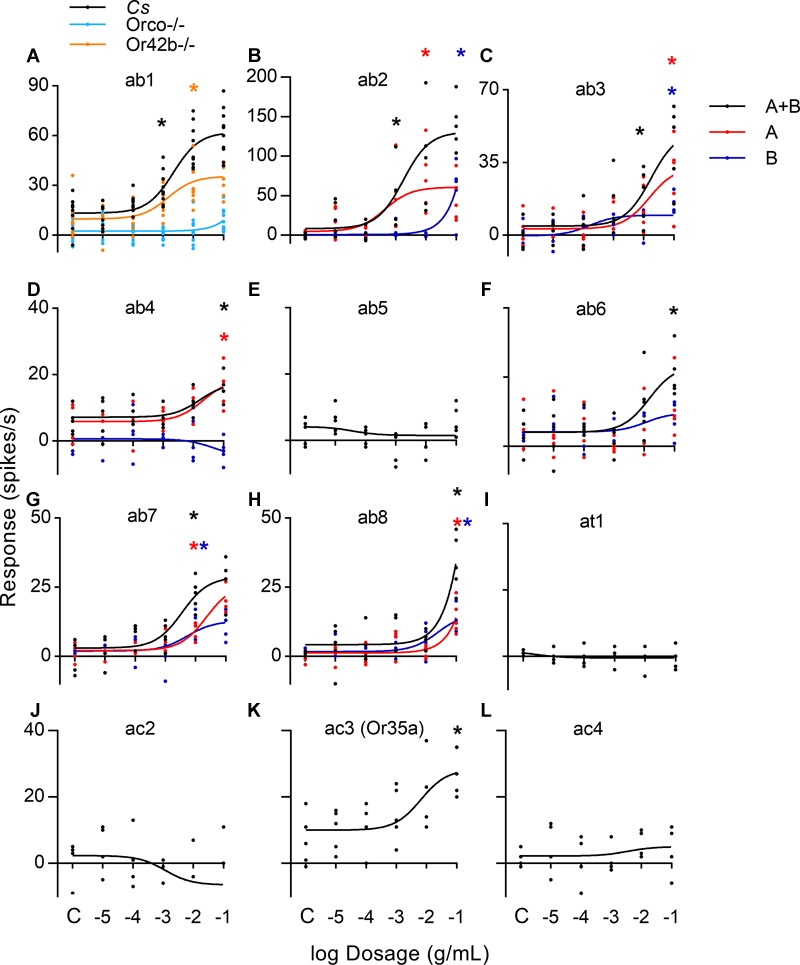
Single sensillum responses of *Drosophila melanogaster* olfactory sensory neurons to coffee furanone. Concentration-response curves for coffee furanone in **A**, Olfactory sensory sensilla ab1 for wild type, *CantonS* (*Cs*) *D. melanogaster*, Or42b-/-, and Orco-/- flies (*n* = 10 each; C = solvent control). **B–L**, Listed olfactory sensilla types for *Cs* flies (*n* = 5 each for ab2-ab8, at1 and ac3; *n* = 4 each for ac2 and ac4). **B–L**, Responses for each sensillum are listed as total sensillum response (black), large (A; red) and/or small (B; blue) spiking neurons. Receptors expressed in each responding neuron type listed in Table 1. ^∗^*p* value < 0.05 at first significant test concentration, paired t-test.

By using the Orco-/- fly strain (Larsson et al., 2004), we were able to silence all Or-expressing OSNs, including ab1 sensilla, which are also the only sensilla that house antennal Grs known to respond to a volatile (carbon dioxide) in a single antennal OSN, ab1c (De Bruyne et al., 2001; Suh et al., 2004) (Figure 2A). The function of other Grs found in the antenna, namely Gr64b and f, is not yet known (Fujii et al., 2015). The loss of response to coffee furanone in ab1 indicates that the antennal Gr21a and Gr63a, responding to carbon dioxide (De Bruyne et al., 2001; Suh et al., 2004), are not likely targets for the compound, but Ors are. Or-specific silencing of ab1 cells expressing Or42b (Hallem et al., 2004) also showed that Orco expression itself is not sufficient for a full response (Figure 2A). Subsequent electrophysiological examination of OSN responses in other sensilla expressing Ors (Figure 2B–I) showed response in nearly all Or-expressing neurons of *Drosophila*, or 15/19 neurons containing Or-expressing cells (*n* = 55) (Figure 2 and Table 1). Ir-expressing neurons did not respond (Figure 2J–L, *n* = 8), apart from sensillum ac3, which also houses an Or-expressing cell (Benton et al., 2009) (*n* = 5). While we observed significant responses from most of the sensilla types, it is important to note that the response intensity varied widely among cell types. Neurons in sensilla ab1 and ab2 exhibited significant responses at a concentration of 10^-3^ g/mL while ab3, ab4, and ab6 – ab8 showed responses at 10^-2^ or 10^-1^ g/mL starting dilutions (Figure 2 and Supplementary Figure 2). These results suggest variable binding affinities for coffee furanone across the receptor types. As implied by our electroantennogram analyses across orders (Figure 1A), OSNs in other species also responded to coffee furanone (Supplementary Figure 3). Combined, these analyses indicate that coffee furanone is detected by Ors of multiple insect species, rather than Grs or Irs.

**Table 1:** List of Ors activated by coffee furanone in this study.

Neuron	Or
ab1a	Or42b
ab1b	Or92a
ab1d	Or10a
ab2a	Or59b
ab2b	Or85a
ab3a	Or22a
ab3b	Or85b
ab4a	Or7a
ab6a	Or13a
ab6b	Or49b
ab7a	Or98a
ab7b	Or67c
ab8a	Or43b
ab8b	Or9a
ac3b	Or35a

To directly compare the promiscuity of our tested compound with other known ligands, we compared responses of Or-expressing neurons for coffee furanone against 2-heptanone, the most broadly detected ligand known for *Drosophila* (Hallem et al., 2004; Münch and Galizia, 2016) at identical concentrations to those used in many previous studies (10^-2^ g/mL). By comparing the spike rates (spikes per sec) normalized to the highest spiking cells, we observed a broader response profile for coffee furanone as compared to 2-heptanone over the same receptor neurons and concentrations (Supplementary Figure 4). Thus, there is no known volatile reported to activate neurons across all of these sensilla at the concentrations tested (Münch and Galizia, 2016). Furthermore, a previous study of the *Drosophila* antenna and antennal lobe (Knaden et al., 2012) using even higher concentrations of 110 compounds (10^-1^ g/mL) did not elicit such broad responses. Additionally, note that for all behavioral and *in vivo* physiological analyses shown here, 10 μl of the listed concentration was eluted onto a filter paper dispenser immediately before the analysis. Stimuli were presented by passing air over this dispenser. As such, the amount reaching the antenna will be much less than the reported initial concentration. This confirms coffee furanone is a unique ligand eliciting neuronal responses across a broad range of receptor types.

**FIGURE 3:**
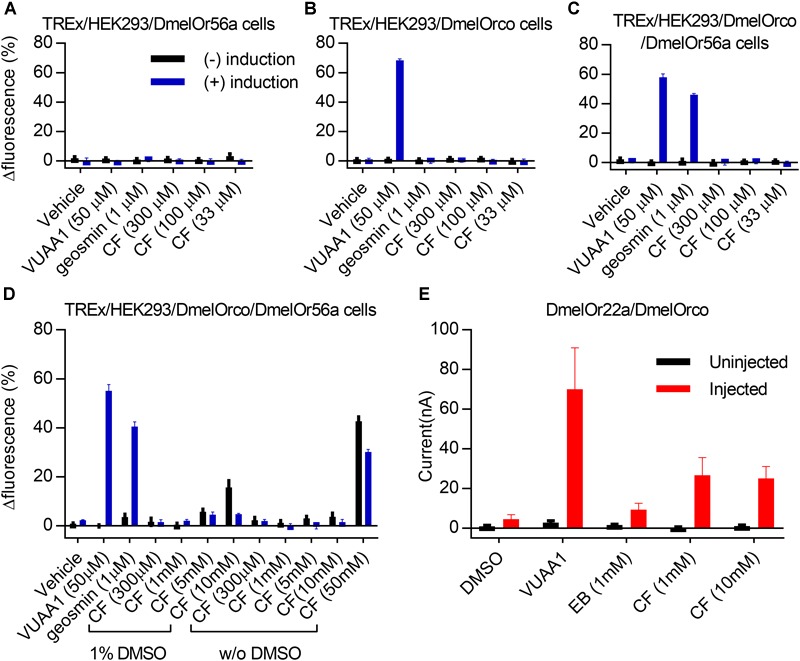
*Drosophila melanogaster* olfactory receptor activation by coffee furanone in heterologous expression systems. DmelOr56a, which is a geosmin-specific *D. melanogaster* receptor, was heterologously expressed in HEK293 cells to study the effect of coffee furanone (CF) on cell lines expressing: **(A)** DmelOr56a alone; **(B)** DmelOrco alone; **(C,D)** DmelOr56a and DmelOrco (*n* = 3 for each). Cell lines induced to express Ors and/or Orco (blue bars) and non-induced control cells (black bars) were treated with vehicle, the Orco-specific agonist VUAA1, geosmin or various doses of CF. Cells expressing **(D)** DmelOr56a/DmelOrco were tested for responsiveness to CF with and without DMSO in order to ensure solubilization of the compound at escalating doses in this assay system. **(E)** DmelOr22a, a broad spectrum receptor with ethyl butyrate (EB) as a primary ligand, was expressed in *Xenopus laevis* oocytes (red bars; black bars represent uninjected controls) and tested for current activation upon stimulation with VUAA1, EB and two concentrations of CF (*n* = 10 for injected, *n* = 10 for uninjected oocytes).

Or-expressing OSNs in three sensilla from *D. melanogaster* did not respond to our compound even at high concentrations: ab5, at1 (Figure 2E,I), and the ab4b OSN (Supplementary Figure 2D). These sensilla house neurons with Ors specifically tuned to pentyl acetate, 2-heptanone and 3-methylthio-1-propanol (Or47a; ab5a), geranyl acetate (Or82a; ab5b), 11-*cis*-vaccenyl acetate (Or67d; at1) (De Bruyne et al., 2001; Hallem and Carlson, 2004; Xu et al., 2005), and geosmin (Or56a; ab4b) (Stensmyr et al., 2012). This lack of response was not a result of the peri-receptor environment, as these sensilla do not necessarily house unique olfactory-binding proteins (Larter et al., 2016). To confirm this lack of response was due to the complex itself and not to other molecules involved in the signal transduction cascade, we heterologously expressed *D. melanogaster* Or56a, responding selectively to geosmin (Stensmyr et al., 2012) both separately and together with Orco proteins in Human Embryonic Kidney (HEK)-293 cell lines (Figure 3A–D). These experiments confirmed that the lack of response in this specialized Or was retained in the heterologous system, and the strong response to the Orco ligand VUAA1 reinforced that coffee furanone does not interact with Orco itself. Conversely, Or22a, a broad spectrum Or from *D*. *melanogaster*, was not effective in our (HEK)-293 lines, and hence we expressed the complex in *Xenopus laevis* oocytes to verify heterologous activity using two-electrode voltage clamp (Zhang and Löfstedt, 2013). A current influx in Or22a-Orco injected oocytes (Figure 3E and Supplementary Figure 5) upon stimulation with coffee furanone confirmed that it interacts directly with the Or22a-Orco complex. Our coupled electrophysiological (EAG and SSR) and heterologous expression studies thus led us to conclude that coffee furanone is a ligand for all but the most specific Or-expressing neurons, and interacts with the Or subunit of the OrX-Orco complex. The lack of response in specialized receptors and other chemoreceptor types further indicates that the observed responses are due to ligand promiscuity and not a non-specific cellular response to high stimulus concentrations.

**FIGURE 4:**
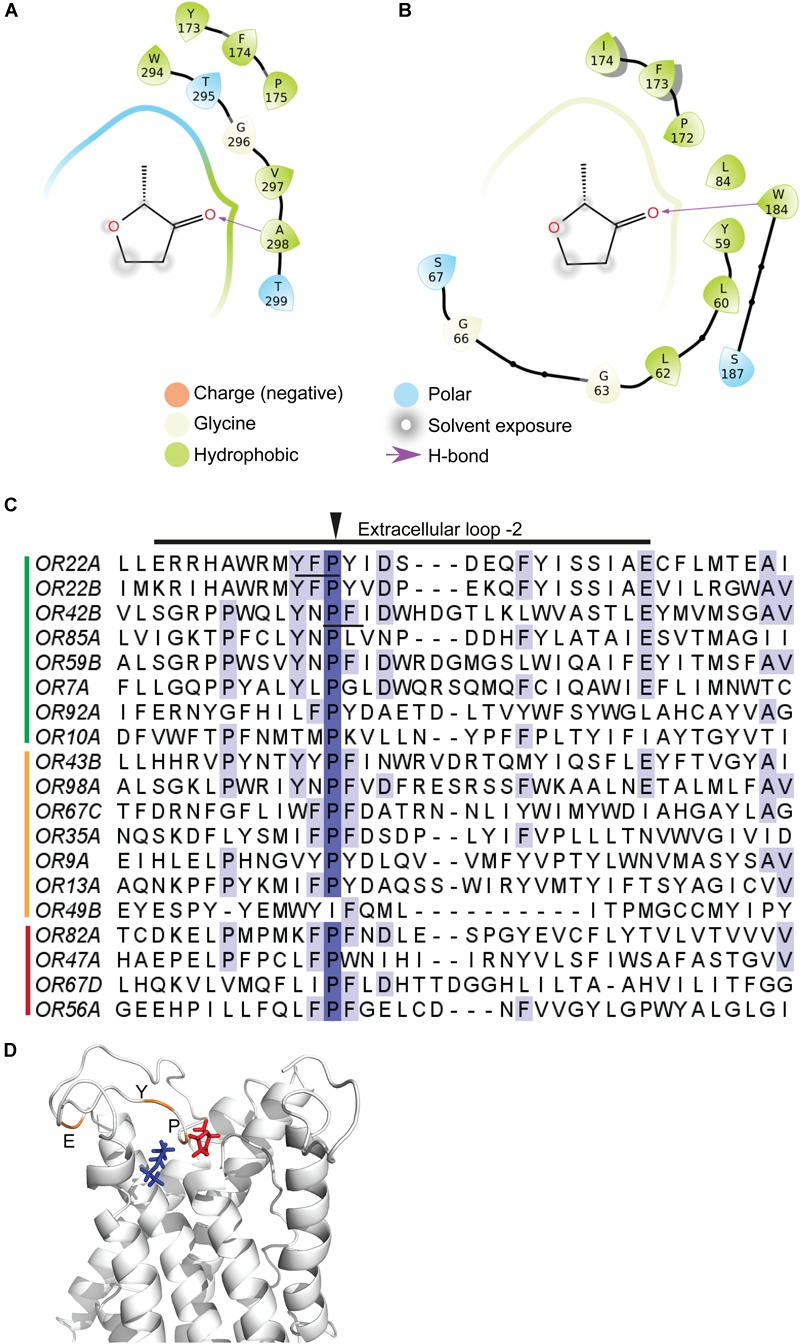
Bioinformatics analysis for interaction of coffee furanone with Or22a and Or42b in *Drosophila melanogaster.* Amino acid residues in interaction within 5Å of coffee furanone as depicted by ligand interaction diagram for pairs **(A)** Or22a-coffee furanone; **(B)** Or42b-coffee furanone; **(C)** Multiple sequence alignment of olfactory receptors highlighting the extracellular loop-2 region. The conserved proline residue found in predicted binding pockets is highlighted by arrow on top. Motif YFP (residues 173-175) and PFI (residues 172-174) in Or22a and Or42b, respectively, are underlined. Green represents Ors expressed in OSNs strongly activated by coffee furanone in our electrophysiology studies; yellow represents Ors in weakly activated OSNs, and red represents Ors in OSNs that are not activated by coffee furanone. **(D)** Evolutionary conserved residues Tyr 173 (Y), Pro 175 (P) and Glu 190 (E) in extracellular loop-2 for Or22a-Or42b class are highlighted as orange in dockpose of Or22a with ligands coffee furanone (red), and ethyl butyrate (blue).

To illustrate the potential of this molecule for understanding chemoreception in this unique receptor family, we chose two broad spectrum Ors – Or22a and Or42b, which were shown to be activated by coffee furanone *in vitro* and/or *in vivo*. We then modeled the three dimensional structures (Šali and Blundell, 1993) for proteins based on the olfactory receptor co-receptor Orco from *Apocrypta bakeri* (Butterwick et al., 2018). Docking was subsequently performed with the Or models only, as this unit has been known to be vital in governing the response of neurons to odorants (Dobritsa et al., 2003) and Orco is not involved directly in odorant binding (Nichols and Luetje, 2010; Leal, 2013). For docking, we focused on the extracellular side of the membrane protein where the ligand would be most likely to interact with the protein. To this end, extracellular loop-2 was modeled based upon I-TASSER (Yang et al., 2014) prediction and intracellular loop-3 was excised in Or42b. We choose one known ligand for each Or for comparison with coffee furanone. Docking protocols with rigid receptors and flexible ligands were carried using the GLIDE suite (Friesner et al., 2006). Our predicted ligand-binding pockets consisted mostly of hydrophobic residues with a few hydrogen bonds (Figure 4A,B and Supplementary Figure 6). A single binding pocket was proposed for both the known ligands and coffee furanone in Or22a and Or42b (Figure 4A,B and Supplementary Figure 6). These predictive results suggest that coffee furanone does not bind at allosteric sites (Figure 4 and Supplementary Figure 6). Furthermore, Or22a and Or42b exhibited a conserved proline residue in motif YXP in these binding pockets (Figure 4C) in extracellular loop (ecl)-2, suggesting possible conserved sites of interaction for odorants. Evolutionary trace analysis also implicated the importance of residues tyrosine, proline, and glutamic acid in extracellular loop -2 (Figure 4D) regions of the Or22a-Or42b class of olfactory receptors (Lua et al., 2016).

The proline residue interacting with coffee furanone and known ligands for Or22a and Or42b is conserved across nearly all responding receptors (Figure 4C), including those whose neurons are not activated by coffee furanone, such as Or56a and Or67d. This implies that this residue cannot be the only potential contributor for receptor activation. To test this prediction, we also modeled ligand-binding pockets for Or56a and Or67d for coffee furanone and its known ligands, geosmin, and 11-*cis*-vaccenyl acetate (cVA), using similar protocols as for Or22a and Or42b. Or56a was predicted to contain two ligand pockets for both coffee furanone and geosmin, neither of which involved ecl-2 or the conserved proline. Meanwhile, Or67d was predicted to interact with coffee furanone as a small part of a much larger binding site for cVA, its known ligand (Kurtovic et al., 2007) (Supplementary Figures 7, 8), which did include ecl-2 and the conserved proline. These predictions suggest that while ecl-2 and the conserved proline could form a base for ligand binding across many of the activated receptors, specialized receptor proteins could utilize alternative regions of the protein as binding sites, or additional proteins. For example, Or67d sensitivity is known to be regulated by sensory neuron membrane protein (SNMPs) (Jin et al., 2008) and odorant binding protein LUSH (Xu et al., 2005).

## Discussion

We have identified a promiscuous small molecule that targets the highly divergent family of ligand-gated ion channels comprising the insect olfactory receptor complex, whose proteins exhibit as little as 10% sequence identities between family members (Benton, 2015). Antennae of insects from several orders detected coffee furanone, and multiple species exhibited either attraction (cockroaches) or repellence (flies and mosquitoes) to this compound in a dose-dependent manner.

What makes coffee furanone unique from other known ligands? While some compounds such as linalool (Raguso, 2016) are detected by a wide range of taxa, they do not necessarily bind to several receptors. Linalool, for example, has a relatively narrow tuning curve across receptors in *Drosophila* (Münch and Galizia, 2016) and is detected by specific receptors in moths (Große-Wilde et al., 2010). Other compounds, such as 2-heptanone, the most broadly detected ligand known for *Drosophila* (Münch and Galizia, 2016), activate a large number of receptors, but still do not approach the tuning breadth of coffee furanone shown here. Coffee furanone is not a key ligand, but rather a unique one, as no other compound stimulates as many neurons at the same concentrations used here. The lack of response in specialized neurons, Orco(-/-) mutants, and those expressing Irs and Grs also indicates that the observed neuronal responses are not a result of non-specific cellular responses. Furthermore, the concentrations of coffee furanone to which the neurons respond are identical to those previously used for other ligands (Hallem et al., 2004; Knaden et al., 2012) that do not elicit such widespread response. Thus, our results suggest that this ligand broadly activates many receptors, and activates chemosensory antennal neurons across many insect species. Finally, this single molecule elicits significant behavioral responses across diverse insect orders. Our results thus indicate that this volatile is a promiscuous ligand activating the variable “OrX” unit of the Or-Orco complex across several flying insect orders.

The broad activity of this ligand across the insect olfactory receptor family provides a unique tool to study how chemoreceptors generate diverse ligand specificities and behavioral activities, as well as how their evolution compares to other multigene families (Benton, 2015). Here we have provided a series of analyses to illustrate the application of this ligand in physiological, heterologous expression, behavioral, and bioinformatic analyses of insect olfaction. For example, our modeling analyses suggest that this molecule could target the same sites as known ligands and allosteric sites are unlikely. This analysis also suggests a conserved proline residue and motif YXP in extracellular loop-2 of Ors whose neurons are highly activated by coffee furanone (Figure 2, 4C). In agreement with our predictions, the extracellular loop-2 region of Or proteins has previously been implicated in ligand binding in mosquitoes and moths, and mutational studies have found proline residues to be critical for function of Ors (Nichols and Luetje, 2010; Xu and Leal, 2013; Hughes et al., 2014). Another study in *Anopheles gambiae* (Ray et al., 2014) found conserved amino acid motif in the N-terminal domain to be functionally important for the detection of phenolic compounds. Our docking studies did not predict N-terminal motifs to be directly involved in binding to our tested compounds. This difference highlights the potential for unique binding sites depending on the ligand examined. As olfactory receptors of more insect species are deorphanized, these molecular predictions regarding both specificity and selectivity can be compared across a wider repertoire of proteins using coffee furanone as a common ligand.

Finally, apart from its significance for understanding the molecular basis of chemoreception, coffee furanone exhibits behavioral activity in multiple insect orders. This compound is also a volatile constituent of many foods, and a common flavoring agent (Food and Agriculture Organization of the United Nations World Health Organization, 2005; World Health Organization, 2006). Consequently, this compound has potential to serve as a safe repellent or attract-and-kill volatile in many contexts including disease vectors like mosquitos, and household pests such as flies and cockroaches, for which it has shown activity in this study. Coffee furanone thus provides a unique tool across a highly divergent family for use in biosensor development, pest control, biomedical applications, and a general understanding of the most specious taxon on Earth.

## Materials and Methods

### Chemicals

The following chemicals were purchased at highest purities available from Sigma Aldrich, Bangalore, India: 2-methyltetrahydro-3-furanone, acetic acid, benzaldehyde, 1-octanol, (*R*)-1-octen-3-ol, ethyl butyrate, ethyl acetate, geranyl acetate, methyl salicylate, methyl laurate, isopentyl acetate, hexanoic acid, 2-methylphenol, geosmin, butyraldehyde, 1,4-diaminobutane, phenyl acetalydehyde, phenylethylamine, pyridine, ammonia solution and mineral oil. 11-*cis*-vaccenyl acetate was purchased from Cayman Chemical Company, Michigan, United States. *Cis*-3-hexenyl acetate, 2,3-butanedione, butyric acid, linalool, and acetophenone were purchased from Fluka, Sigma-Aldrich, Bangalore, India. The compound 1-hexanol was purchased from TCI, nonanal was purchased from Acros Organics. Propionic acid was obtained as a gift from the Max Planck Institute for Chemical Ecology, Jena, Germany. Fluo-4AM was purchased from Life Technologies, Stockholm, Sweden.

### *In vivo* Studies

Chemicals for chemoreception studies were dissolved in mineral oil in a serial dilution ranging from 10^-1^ to 10^-5^ g/mL. Chemical delivery has been described earlier (Tait et al., 2016).

Animal preparation for electroantennography (EAG) and single sensillum recording (SSR) for *Drosophila melanogaster* and *Apis dorsata* has been described previously (Pellegrino et al., 2010; Tait et al., 2016). EAGs with other insects were conducted using excised heads or antennae connected to a forkhead electrode (Ockenfels Syntech, Kirchzarten, DE) using electrode gel (Parker Laboratories, INC, NJ, United States). Data acquisition was performed using EAG2000 software (Ockenfels Syntech, Kirchzarten, DE). Protocol for SSR was as described (Pellegrino et al., 2010; Tait et al., 2016) for all species tested (*Drosophila melanogaster, Rhagoletis pomonella, and Helicoverpa armigera*). Stimuli were presented as 0.5 s pulses at 0.5 L/min using a custom built stimulus delivery setup as described in Tait et al. (2016). Syntech intelligent data acquisition controller (IDAC-4) and Autospike software (Ockenfels Syntech, Kirchzarten, DE) were used for SSR data acquisition.

### *In vitro* Studies

Human Embryonic Kidney 293 (HEK293) cells were used to test the effect of coffee furanone on dipteran olfactory receptor complexes as previously described (Corcoran et al., 2014). Briefly, open reading frames (ORF) coding for *Drosophila melanogaster* (Dmel) Orco was ligated into pcDNA4/TO (Life Technologies) and ORFs for DmelOr56a was ligated into pcDNA5/TO (Life Technologies) and used to generate HEK293 cell lines with stable, regulated expression of: (1) DmelOrco alone, (2) DmelOr56a alone, and (3) DmelOrco and DmelOr56a. The stably-expressed plasmids within the cell lines express olfactory receptors under the regulation of the “Tet-Repressor” that constitutively represses expression of transfected constructs until an induction agent is added to the culture medium. Cell lines were tested for responsiveness to vehicle (i.e., solvent in which compounds were dissolved), VUAA1 (an Orco-specific agonist), ligands specific for each olfactory receptor (geosmin for DmelOr56a), and various doses of coffee furanone. Changes in intracellular calcium levels were measured using the calcium-sensitive fluorophore Fluo-4AM (Life Technologies) and an Omega FluoStar plate reader, and responses to compounds were expressed as a percent increase from baseline fluorescence as previously described (Corcoran et al., 2014).

For the expression in *Xenopus* oocytes, the ORFs for *D. melanogaster* Or22a and the co-receptor Orco were ligated into the expression vector pCS2+. The linearized recombinant plasmids were used as template to synthesize the cRNAs with mMESSAGE mMACHINE kit (Thermo Fisher Scientific). The oocytes were collected from adult female *X. laevis* (purchased from Xenopus Express France, Vernassal, Haute-Loire, France). After being pre-treated with 1.5 mg/mL collagenase (Sigma-Aldrich Co., St. Louis, MO, United States), each oocyte was co-injected with 50 ng of DmelOr22a and DmelOrco mRNA. The oocytes were then incubated at 18 ± 1°C for 3–5 days and the whole-cell inward currents at the holding potential of -80 mV were recorded by two-electrode voltage clamp coupled with a TEC-03BF amplifier (npi electronic GmbH, Tamm, Germany). The uninjected oocytes were used as negative control. The tested compounds were dissolved in dimethyl sulfoxide (DMSO) (Sigma-Aldrich Co., St. Louis, MO, United States) as stock solution and diluted to indicated concentrations with 0.1% of DMSO by Ringers’ buffer. Each recorded oocyte was successively perfused with the compounds at a rate of 2 mL/min with extensive washing in the intervals with Ringer’s buffer until recovery from the stimulation. The currents are recorded as the maximum magnitude from the pre-stimulus baseline. Data were collected and analyzed by Cellworks software (npi electronic GmbH, Tamm, Germany).

### Behavioral Assays

Y-tube choice assays were performed with *Drosophila melanogaster, Aedes aegypti*, and *Blattella germanica*. A borosilicate Y-tube was placed in a vertical (for *D. melanogaster*) or horizontal position (for mosquitoes and cockroaches). For stimulus delivery, 10 μl of chemical dilution and solvent was aliquoted to filter paper disks (diameter: 2.25 cm) suspended in 1 mL pipette tip and attached to the arms of the Y-tube. Humidified air was pushed into the Y-tube using a Syntech stimulus controller (CS-55-V2, Ockenfels Syntech, Kirchzarten, DE) at a velocity of 0.5–0.6 m/s. Insects were tested individually. For *Drosophila* and mosquitoes, the Y-tube had an internal diameter of 16 mm, common arm length 150 mm, angle 45° and side arm length 200 mm. For German cockroaches, the Y-tube had an internal diameter of 27 mm, common arm length 180 mm, angle 45° and side arm length 200 mm.

Response index (RI) was calculated for each species using following formula:

RI= (Number of individuals which go toward chemical arm -Number of individuals which go toward solvent        control arm)/total number of responding individuals.

### Bioinformatic Analyses

#### Transmembrane Domain Prediction

Transmembrane domains for each of the three Ors were predicted using eight different methods: TMPRED (Hofmann and Stoffel, 1993), MEMSAT3 (Jones, 2007), MEMSAT-SVM (Nugent and Jones, 2009), PHDhtm (Rost et al., 1995), HMMTOP(Tusnády and Simon, 2001), TMHMM (Krogh et al., 2001), PHOBIUS (Käll et al., 2004) and PolyPhobius (Käll et al., 2005), and consensus of at least four methods was taken for each domain, except for the transmembrane domain 7 in Or56a for which only a single method predicted the domain, and for TM7 in Or67d where consensus was not considered (Table 2).

**Table 2:** Consensus predictions of transmembrane helices for Ors.

Olfactory Receptor	TM1	TM2	TM3	TM4	TM5	TM6	TM7
Or22a	47–71	84–108	140–164	191–215	265–290	294–318	363–387
Or42b	43–67	82–102	139–161	189–215	267–291	297–319	360–381
Or56a	44–64	76–97	137–162	203–229	290–315	323–342	389–413
Or67d	41–65	70–93	134–161	182–215	267–292	295–318	361–389

#### Homology Modeling

The olfactory receptor co-receptor, Orco, structure from *Apocrypta bakeri* was used as template for insect Ors (Butterwick et al., 2018). This template protein shares low sequence similarity with selected olfactory receptors [Or22a – 39%, Or42b – 37%, Or56a – 50%, Or67d – 47%, PRALINE (Pirovano et al., 2008) alignment]. The structure was obtained from Protein data bank (Berman et al., 2000), PDB id – 6C70 at a resolution of 3.5 Å.

**FIGURE 5:**
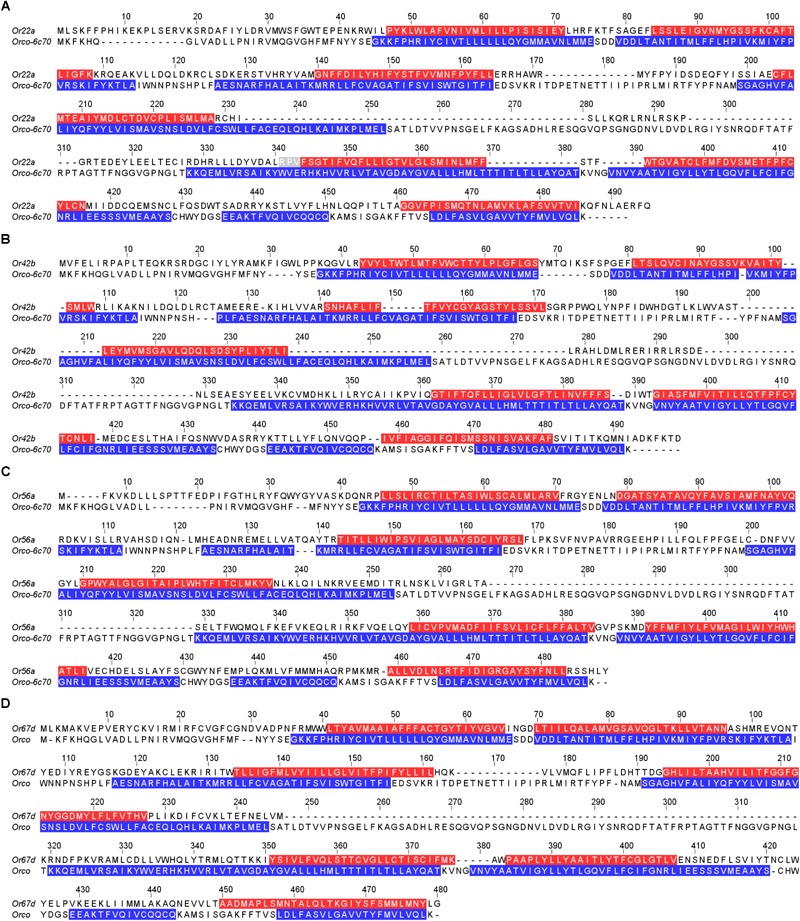
Transmembrane helix region alignment with template for selected *Drosophila melanogaster* olfactory receptors. Alignment of query olfactory receptors and template Orco- 6c70 in **(A)** Or22a; **(B)** Or42b; **(C)** Or56a and **(D)** Or67d. Transmembrane regions highlighted in violet (for query sequence) and in pink (for template sequence).

Pairwise sequence alignment was performed between template and Or using PRALINE server, and manually annotated to align transmembrane regions of both proteins (Figure 5). This alignment was used as input to MODELLER 9.20 (Šali and Blundell, 1993) for homology based modeling. For extracellular loop-2 (ecl-2), secondary structures were predicted by I-TASSER (Yang et al., 2014) and PSIPRED (Jones, 1999). I-TASSER models were used as template for modeling extracellular loop-2. In case of Or42b, Intracellular loop (icl)-3 is very long and pdb 6C70 does not have icl-3, so this loop was excised while modeling Or42b.

20 models were generated for each Or, out of which top energy models based on low Discrete optimized protein energy (DOPE) score in MODELLER were validated using PROCHECK (Laskowski et al., 1993) and ProSA (Wiederstein and Sippl, 2007). More than 98% of residues of models were in allowed regions of the Ramachandran plots and *z*-scores were in permeable limits (Table 3). The residues that were in disallowed regions belonged to loop regions in the models.

**Table 3:** Dope score as given by MODELLER, and Ramachandran plot statistics as validated using PROCHECK with *Z*-scores for models of selected Ors.

Olfactory Receptor	Dope score	Percentage of residues in most favored region	Percentage of residues in additionally allowed region	Percentage of residues in generally allowed region	Percentage of residues in disallowed regions	*Z*-score
Or22a	-44672.320	87.0	9.8	2.7	0.5	-0.33
Or42b	-45747.878	88.1	8.8	2.5	0.6	0.25
Or56a	-56171.269	93.3	5.2	1.3	0.3	-4.95
Or67d	-50310.765	91.6	7.0	0.6	0.8	-4.04

#### Molecular Docking

The best models were selected for Or22a, Or42b, Or56a, and Or67d and used for further docking studies. Docking and associated analysis was performed using Schrodinger’s Maestro (Schrödinger Release 2018-1: Maestro, Schrödinger, LLC, New York, NY, 201) and GLIDE suites (Friesner et al., 2006) (**Schrödinger Release 2016-4**: Glide, Schrödinger, LLC, New York, NY, 2016.). Receptor models were first prepared within a lipid bilayer with energy minimization. SiteMap (Halgren, 2009) predictions were then used to predict putative binding pockets. Receptor grids were placed on the predicted sites in the extracellular regions. A semi-flexible docking protocol where the receptor was rigid and the ligand was flexible used GLIDE-suite for extra precision docking (Friesner et al., 2006). Ligands were prepared using Ligprep. Coffee furanone and one known ligand per receptor was chosen for the study. Dockposes with lowest dockscores (Table 4) were chosen for further study.

**Table 4:** Dockscores of ligand poses.

Olfactory	Ligand	Dockscore
**Receptor**		
Or22a	Ethyl butyrate (EB)	-3.005
Or22a	Coffee furanone (CF)	-3.173
Or42b	Ethyl acetate (EA)	-3.732
Or42b	Coffee furanone (CF)	-2.802
Or56a – Pocket 1	Geosmin	-2.881
Or56a – Pocket 1	Coffee furanone	-2.166
Or56a – Pocket 2	Geosmin	-3.583
Or56a – Pocket 2	Coffee furanone	-3.811
Or67d	11-*cis*-vaccenyl acetate (cVA)	-5.192
Or67d	Coffee furanone	-3.357

##### Ligands used in study

Structure files for ligands ethyl acetate (PubChem CID: 8857), ethyl butyrate (PubChem CID: 7762), geosmin (PubChem CID: 29746), and coffee furanone (PubChem CID: 18522) were obtained from PubChem (Kim et al., 2016). 11-*cis*-vaccenyl acetate (cVA) coordinates were obtained from Protein data bank (pdb) file 2GTE.

#### Multiple Sequence Alignment

Sequences for olfactory receptors were obtained from Uniprot (The UniProt Consortium, 2017). Alignment was carried by Clustal Omega (Sievers et al., 2011) and viewed using Jalview (Waterhouse et al., 2009).

#### Evolutionary Trace Analysis

Evolutionary trace analysis was performed using the UET server (Lua et al., 2016). Sequences of all olfactory receptors in *Drosophila melanogaster* and sequences of olfactory receptors belonging Or22a-Or42b class (Nagarathnam et al., 2014) were used to compare amino acids specific for this class. We reported the residues specific to extracellular regions only (Figure 4D), residues in other parts of the protein (i.e., intracellular and transmembrane) are not mentioned.

### Statistical Analyses

We used Chi-square analysis for Y-tube behavioral data.

For SSR analysis, responses were calculated as:

Difference in number of spikes = number of spikes for 1 s post stimulus - number of spikes for 1 s pre-stimulus.

Regression lines for SSR were fitted using nonlinear method (GraphPad Prism software). Significance was tested using *t*-test for different groups.

Figures were made using GraphPad prism software, California, United States and Adobe Illustrator CS5, California, United States.

## Author Contributions

SB and SO conceived the idea, planned the experiments, analyzed, and interpreted the results. SB and SO wrote the manuscript. SB performed the chemoreception analyses, designed the behavioral assays, analyzed, and interpreted the results. PP performed behavior assays on *Drosophila melanogaster*. UKP performed behavior assays on *Aedes aegypti*. RK performed behavior assays on *Blattella germanica*. JC, SB, and CL performed the HEK cell assays and analyzed the data; D-DZ performed the heterologous expression in oocytes and two-electrode voltage clamp study. SB, SO, and RS designed and analyzed the modeling and docking studies. SB carried out modeling and docking experiments. All the authors reviewed the manuscript.

## Conflict of Interest Statement

The authors declare that the research was conducted in the absence of any commercial or financial relationships that could be construed as a potential conflict of interest.
