# Supplementary material for: A Functional Agonist of Insect Olfactory Receptors: Behavior, Physiology and Structure
Source: Front Cell Neurosci. 2019 Apr 29;13:134. doi: 10.3389/fncel.2019.00134 (PMC6501728; doi:10.3389/fncel.2019.00134)
Supplement: Supplementary file 1 [file Data_Sheet_1.pdf]

Supplementary Figure Legends

Supplementary Figure 1. Electroanntenogram response of Orco-/- strain of *Drosophila melanogaster* to coffee furanone

Stimulus is presented at a concentration of 10^{-1} g/mL for 0.5 s at an airflow of 0.5 L/min and is denoted by red bar on EAG curve.

Supplementary Figure 2. Olfactory detection of Coffee furanone by olfactory neurons

Representative SSR traces from olfactory neurons in *Drosophila melanogaster* showing responses to known ligands (concentration of 10^{-4} g/mL for ab1-3 and 10^{-3} g/mL for others) and lowest significant response to coffee furanone (concentration 10^{-3} to 10^{-1} g/mL as indicated in Figure 2 by black asterisks). Recordings are from 1 s before stimulus and 3.5 s after stimulus. Stimulus is presented for 0.5 s and is depicted by red line.

Supplementary Figure 3. SSR representative responses for various species to Coffee furanone

(A) *Helicoverpa armigera*, and **(B)** *Rhagoletis pomonella* responding to coffee furanone (10^{-1} g/mL) listed known odorants dissolved in mineral oil. Hexane is presented at a concentration of 10^{0} g/mL as in Tait et al., 2016. Recordings are from 1 s before stimulus and 2.5 s after stimulus. Stimulus is presented for 0.5 s and is depicted by red bar.

Supplementary Figure 4. Comparison of normalized responses to coffee furanone and 2-heptanone in *Drosophila melanogaster*

Normalized responses for **A,** coffee furanone and **B,** 2-heptanone at a concentration of 10^{-2} for all wild type antennal *D. melanogaster* receptor neurons reported with electrophysiological response to 2-heptanone in DoOR2.0 (n = 10; Hallem et al., 2004; Münch and Galizia, 2016). Spike rates

per neuron type are normalized to the highest response for each odor and listed according to receptor expressed in that neuron.

Supplementary Figure 5. *In vitro* detection of coffee furanone

Representative current traces of oocytes **(A)** from trial 1 and **(B)** trial 2, expressing DmelOr22a and DmelOrco; and uninjected controls upon stimulation with dimethyl sulfoxide (DMSO), ethyl butyrate (EB), and two different concentrations of coffee furanone (CF) and ethyl hexanoate (EH; trial 2 only)

Supplementary Figure 6. Ligand interaction diagram for Or22a and Or42b *Drosophila melanogaster* olfactory receptor models with known ligands

Amino acid residues in interaction within 5Å of known ligands as depicted by ligand interaction diagram for pairs **(A)** Or22a- ethyl butyrate; **(B)** Or42b-ethyl acetate.

Supplementary Figure 7. Ligand interaction diagram for Or56a and Or67d *Drosophila melanogaster* olfactory receptor models

Amino acid residues in interaction within 5Å of ligand as depicted by ligand interaction diagram for **(A),** Or56a- coffee furanone- pocket 1; **(B)** Or56a- geosmin- pocket 1; **(C)** Or56a- coffee furanone- pocket 2; **(D)** Or56a- geosmin- pocket 2; **(E)** Or67d- coffee furanone and **(F)** Or67d-11-cis-vaccenyl acetate (cVA). The figure was obtained using the "Ligand Interaction Diagram" of the GLIDE software (Schrödinger Release 2013–1:, version 2.6, Schrödinger, LLC, New York, NY, 2013).

Supplementary Figure 8. Snakeplots for *Drosophila melanogaster* olfactory receptor structures

Snakeplots highlighting residues in purple predicted to interact with coffee furanone in **(A)** Or22a; **(B)** Or42b; **C,** Or56a –Pocket 1; **D,** Or56a –Pocket 2 and **E,** Or67d. Snakeplots made using "Johns S.J., TOPO2, Transmembrane protein display software, http://www.sacs.ucsf.edu/TOPO2/"

Legends for additional supplementary files

Supplementary File 1: or22a.pdb - Coordinate file for Or22a modeled structure

Supplementary File 2: or42b.pdb - Coordinate file for Or42b modeled structure

Supplementary File 3: or56a.pdb - Coordinate file for Or56a modeled structure

Supplementary File 4: or67d.pdb - Coordinate file for Or67d modeled structure

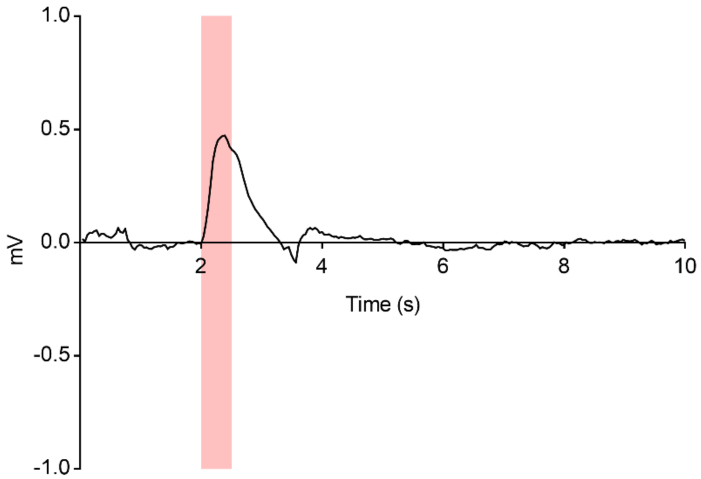

Supplementary Figure 1

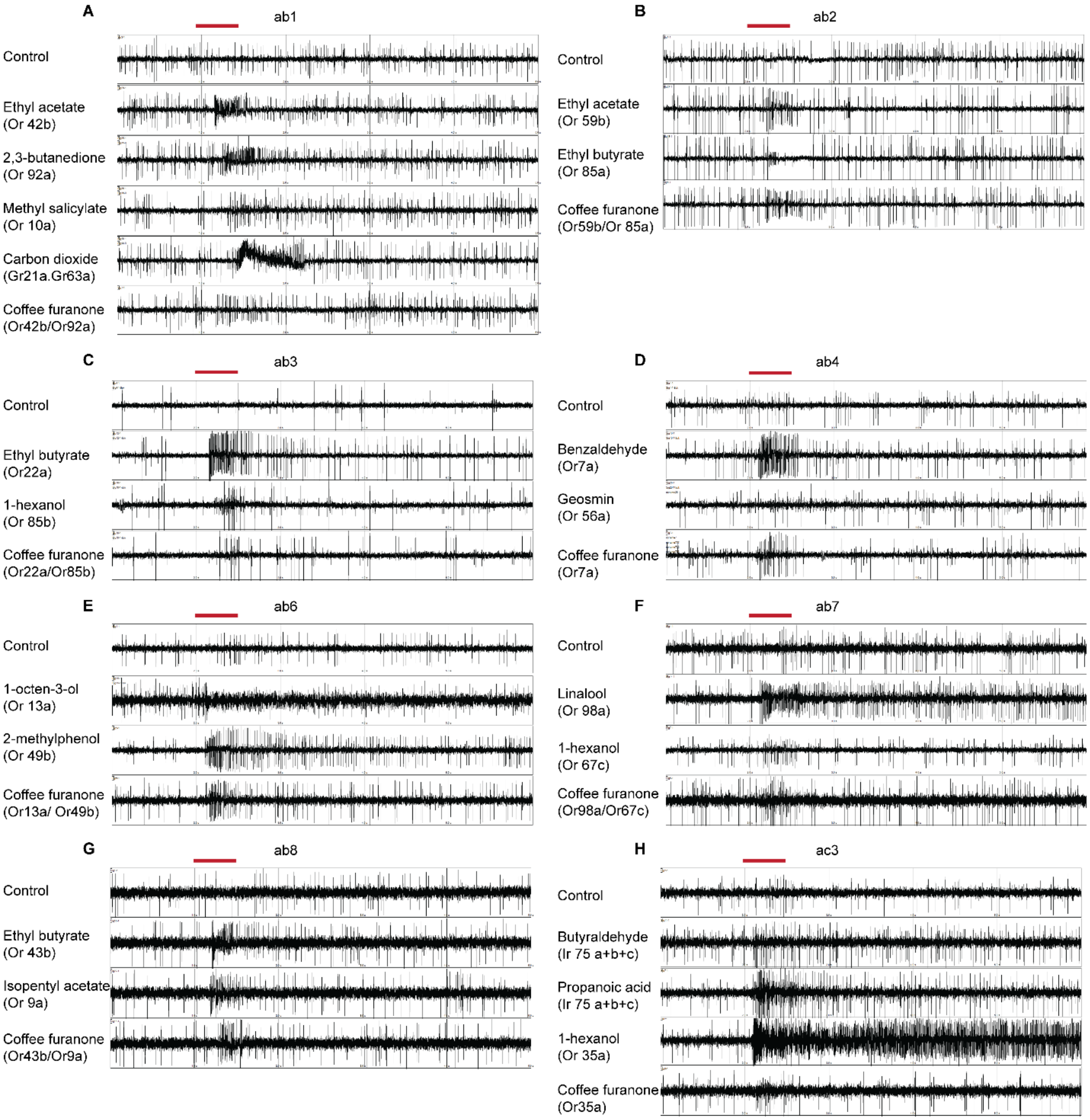

Supplementary Figure 2

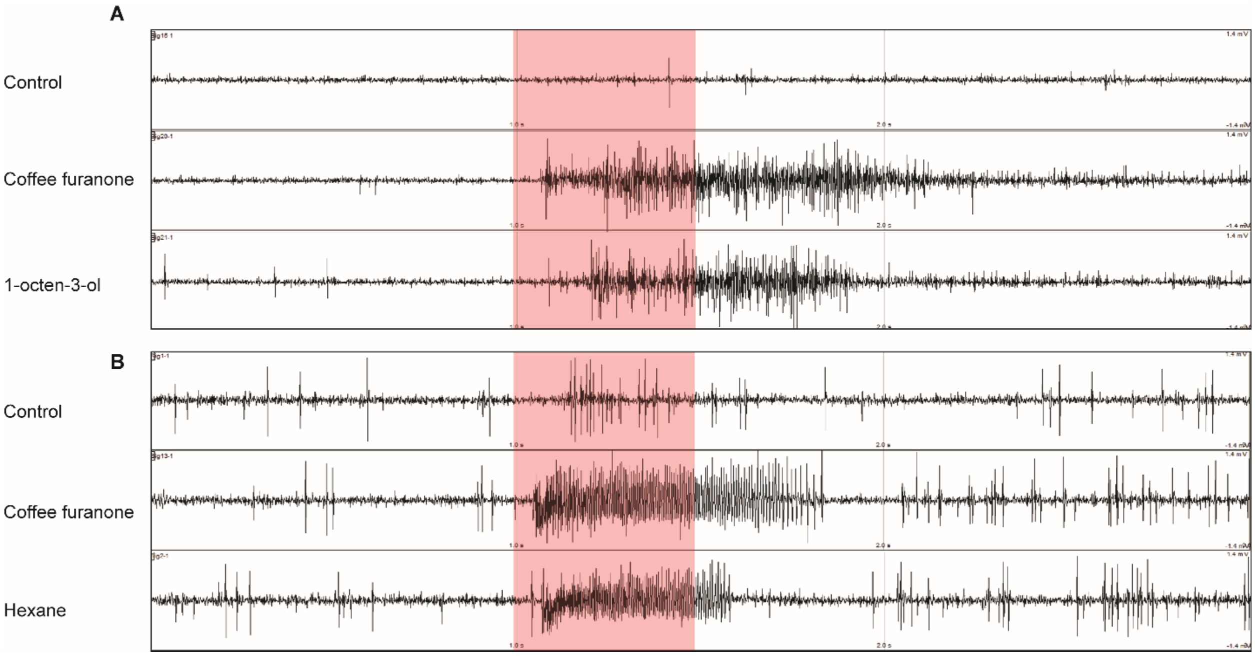

Supplementary Figure 3.

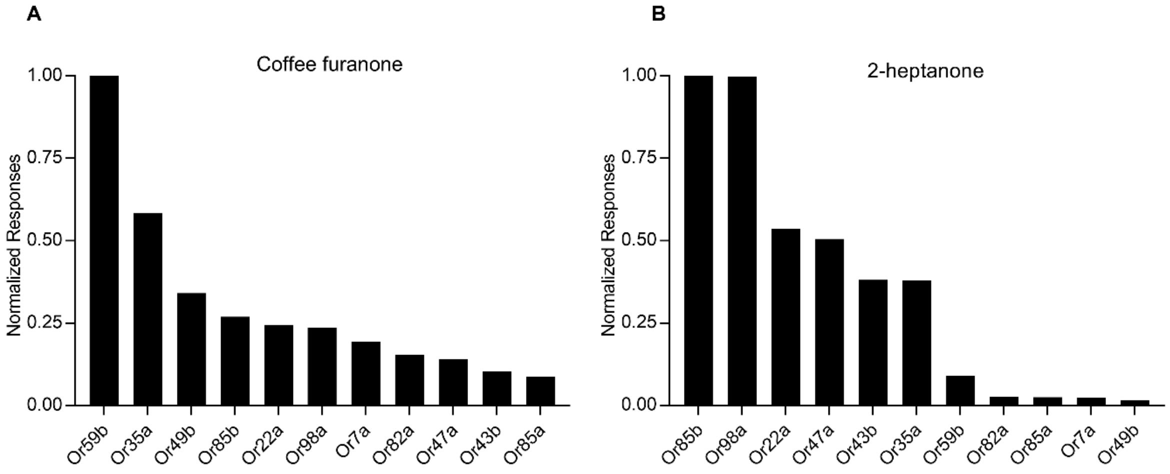

Supplementary Figure 4.

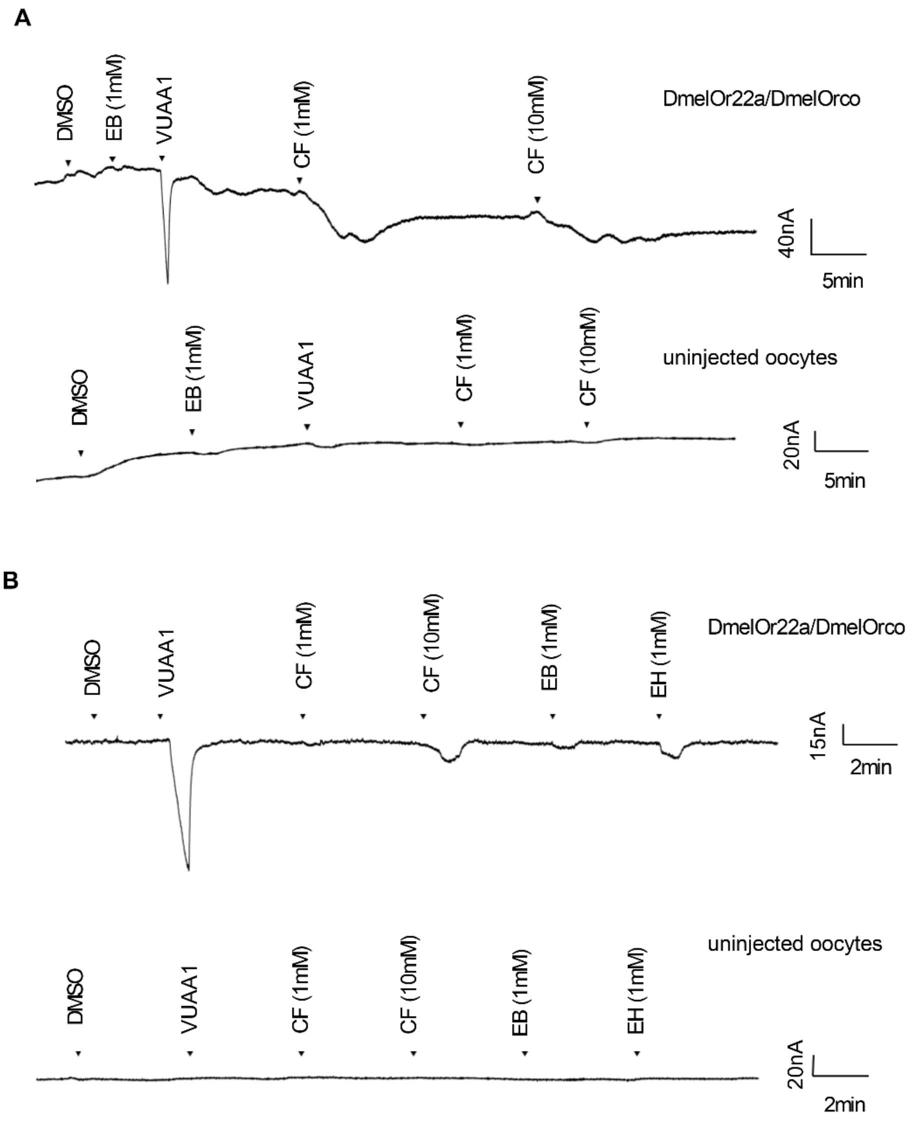

Supplementary Figure 5.

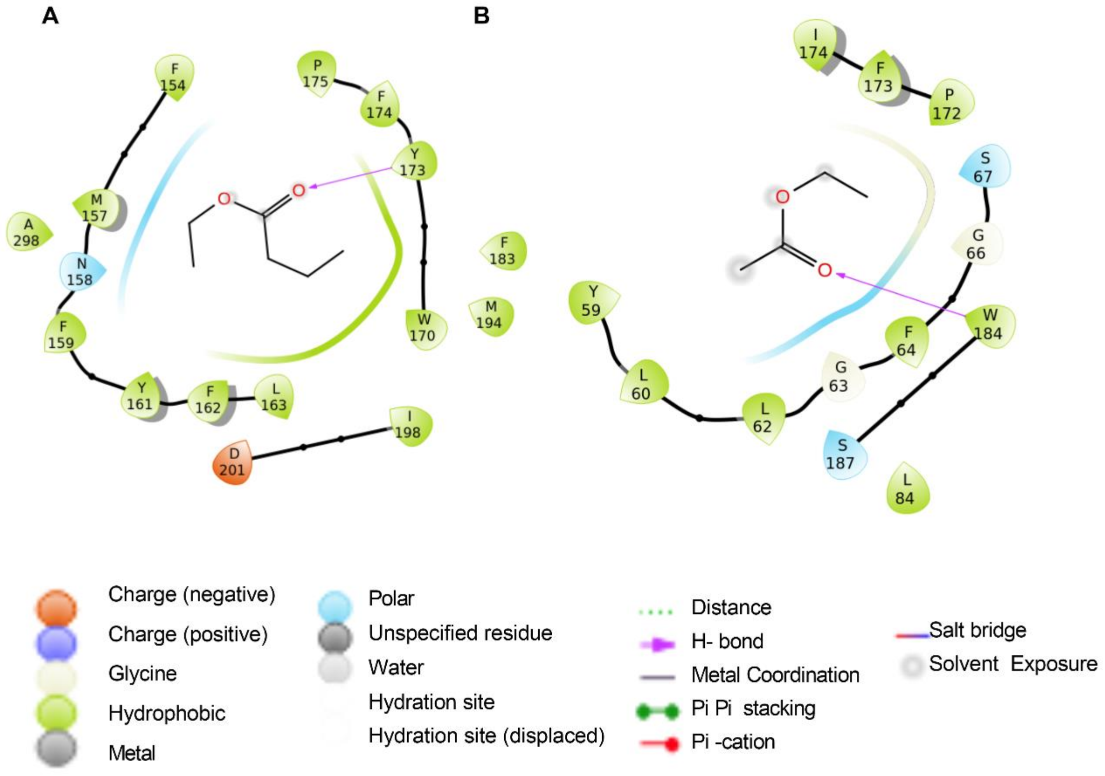

Supplementary Figure 6.

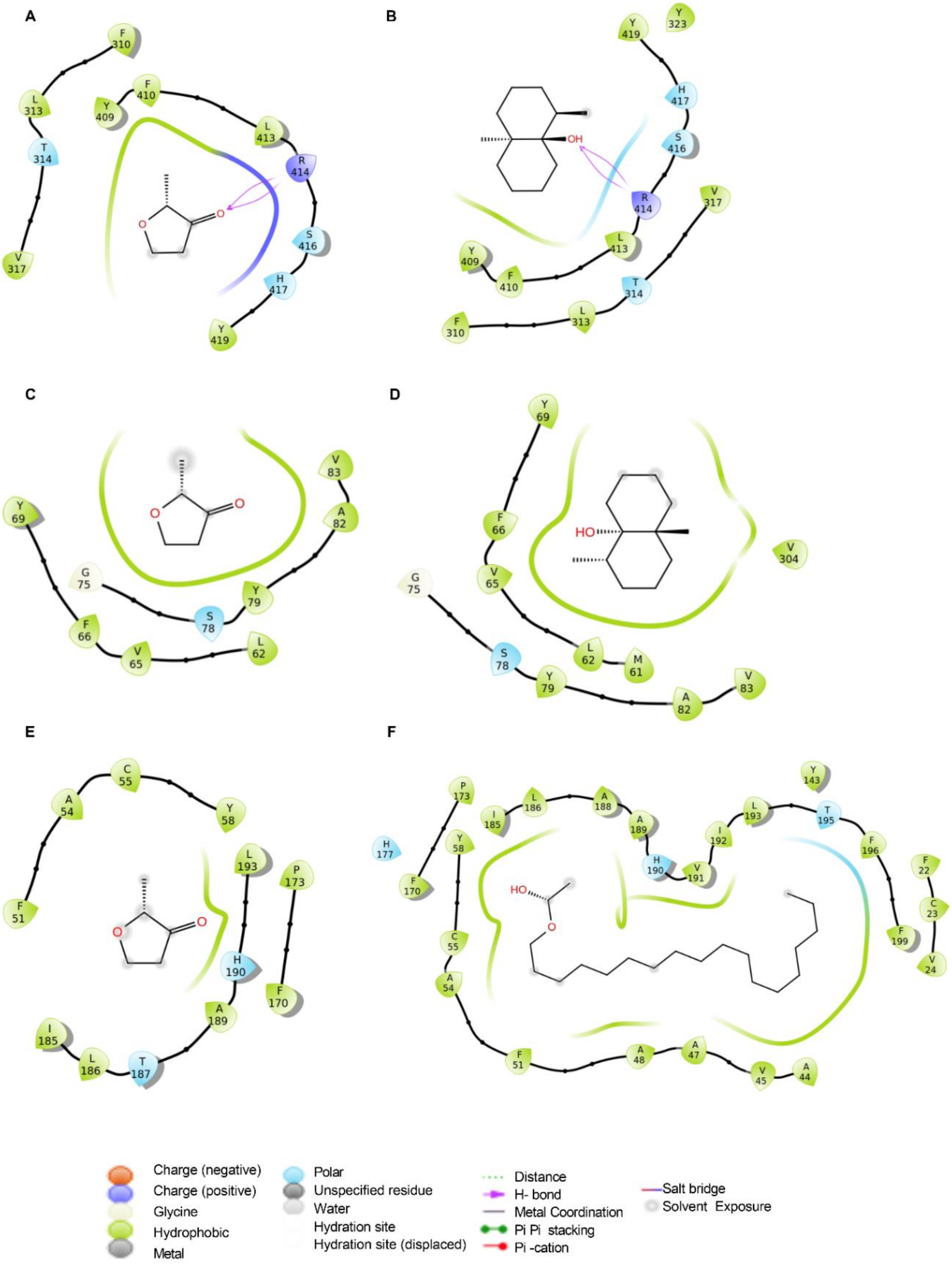

Supplementary Figure 7.

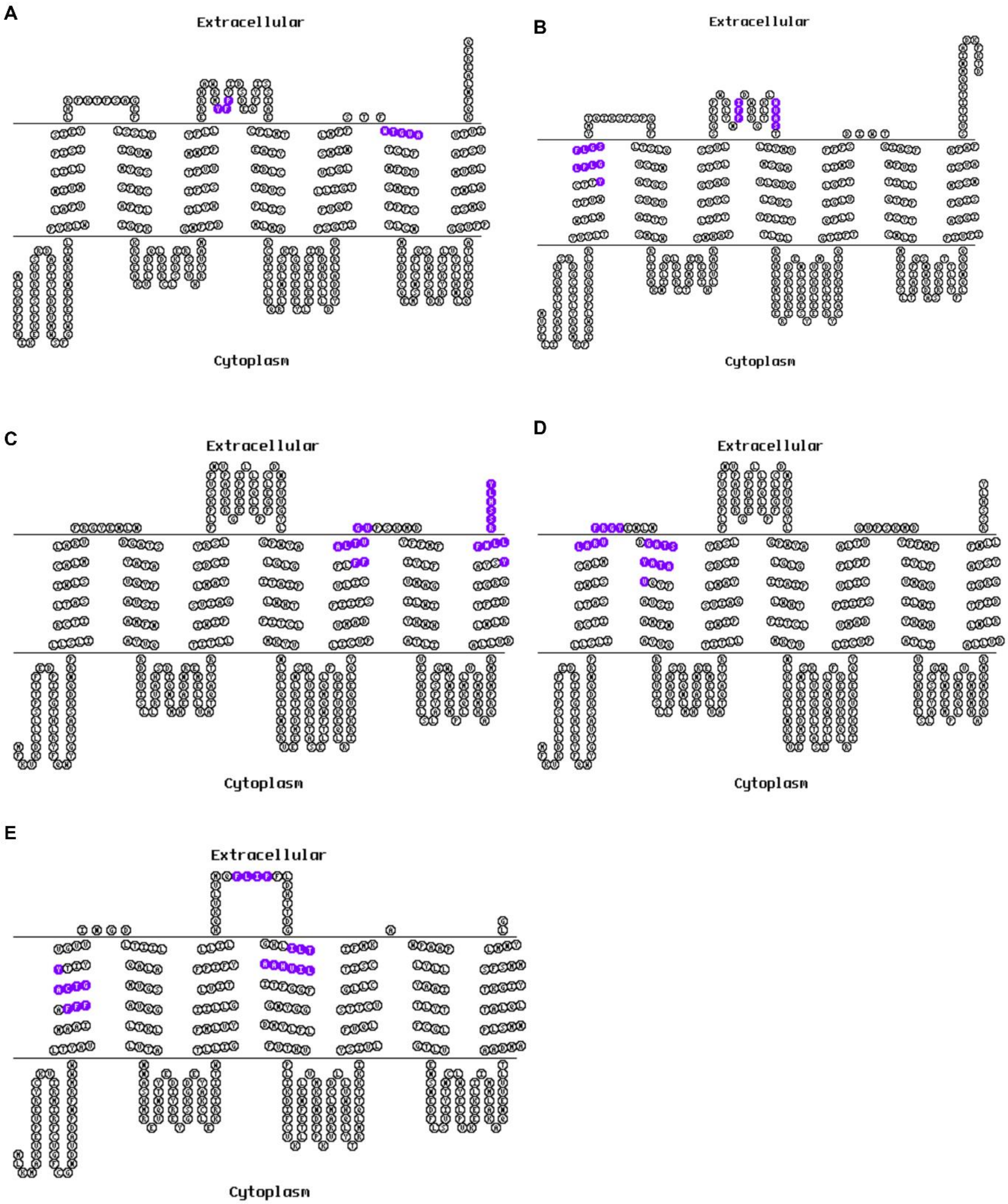

73

74 **Supplementary Figure 8.**
